# Dry eye disease treatment improves subjective quality-of-life responses in patients with AMD, independent of disease stage

**DOI:** 10.1371/journal.pone.0318733

**Published:** 2025-02-06

**Authors:** Nehal Nailesh Mehta, Ines D. Nagel, Akshay Agnihotri, Anna Heinke, Lingyun Cheng, Dirk-Uwe Bartsch, William R. Freeman, Maria-Laura Gomez

**Affiliations:** 1 Jacobs Retina Center, La Jolla, California, United States of America; 2 Viterbi Family Department of Ophthalmology and Shiley Eye Institute, University of California San Diego, La Jolla, California, United States of America; 3 Division of Ophthalmology Informatics and Data Science, Viterbi Family Department of Ophthalmology and Shiley Eye Institute, University of California San Diego, La Jolla, California, United States of America; Icahn School of Medicine at Mount Sinai, UNITED STATES OF AMERICA

## Abstract

**Purpose:**

To determine the impact of severity of age-related macular degeneration (AMD) on subjective treatment response in patients treated for dry eye disease.

**Methods:**

A total of 203 eyes diagnosed with evaporative dry eye disease (DED) due to meibomian gland dysfunction were treated using the LipiFlow or MiBoFlo systems. From this cohort, 40 eyes with stable dry AMD (early, intermediate, or late stages) were included. Each participant completed the Ocular Surface Disease Index (OSDI) and Standard Patient Evaluation of Eye Dryness Questionnaire (SPEED) before treatment and at a 6-month follow-up. Changes in questionnaire scores were analyzed using one-way analysis of variance (ANOVA) to assess differences between AMD severity groups.

**Results:**

Improvement in SPEED and OSDI scores, including vision related OSDI scores were observed across all AMD stages, with no significant differences between groups (p<0.05).

**Conclusion:**

Managing DED improved quality of life (QOL) in patients with AMD, regardless of retinal disease severity. This highlights the importance of treating coexisting ocular surface conditions to enhance patient outcomes, even in the presence of significant maculopathy.

## Introduction

Dry eye disease (DED) is one of the most common chronic ocular complaints presented to the Ophthalmic clinic. The prevalence worldwide is high, and ranges from 5 to 50 percent in different parts of the world [1]. Patients present with a range of symptoms like ocular surface irritation, itching, redness, watering, stinging pain, foreign body sensation and intermittent visual blurring [2]. These symptoms often result in difficulty driving, working on a computer, watching television, and reduced reading speed [3]. Thus, DED can negatively affect quality of life (QOL) and visual function [4].

Dry eye has been defined by the Tear Film and Ocular Surface Society (TFOS) in Dry Eye Workshop II (DEWS II) as a multi-factorial disease of the ocular surface characterized by a loss of homeostasis of the tear film, and accompanied by ocular symptoms, in which tear film instability and hyperosmolarity, ocular surface in-flammation and damage, and neurosen-sory abnormalities play etiological role [5]. The tear film comprises three layers—mucin, aqueous, and lipid—spanning from the corneal surface to the air interface [6]. Secreted mainly by the lacrimal glands, the aqueous layer of tear film provides oxygen and nutrients to the cornea [6]. The lipid layer is secreted by the meibomian glands and plays an important role in preventing excessive evaporation of tears from the eye [7]. DED is classified as either aqueous-deficient or evaporative [8]. Aqueous–deficient DED is a result of lacrimal gland dysfunction, while evaporative DED, present in over 85% of cases, arises from meibomian gland dysfunction, leading to a deficient lipid layer and excessive tear evaporation [8, 9].

Diagnosis of DED commonly involves questionnaires assessing symptoms, as well as clinical evaluations, such as slit-lamp examination, ocular surface staining (fluorescein, lissamine green, or rose bengal), tear film breakup time (TBUT), Schirmer test, and meibomian gland function assessment using interferometry [10]. Ocular Surface Disease Index (OSDI) and Standard Patient Evaluation of Eye Dryness Questionnaire (SPEED) are two of the commonly used quality-of-life questionnaires used to assess patients with dry eye disease [11, 12]. The OSDI questionnaire consists of questions relating to 5 ocular symptomatic domains, 4 vision-related domains, and 3 domains related to environmental triggers [13]. On the other hand, the SPEED questionnaire focuses on the presence, frequency, and severity of four common (dryness/ grittiness/ scratchiness; soreness/ irritation; burning/ watering and eye fatigue) subjective symptoms experienced by patients [12, 14].

Common DED treatments include over-the-counter lubricants to supplement the aqueous or lipid tear film layers [15–17]. Newer therapies like the LipiFlow Thermal Pulsation System (LipiFlow, Tear Science, Johnson and Johnson, Santa Ana, Ca) and the MiBoFlo Thermoflo treatment system (MiBo Medical, Dallas, TX) have been shown by us and others to improve meibomian gland function and thus improve the lipid layer quality of the tear film, providing long term relief (up to 6 months) to patients with evaporative dry eye [18–22]. These heat-based therapies deliver heat to the meibomian glands which softens the inspissated lipids and enables their extrusion by using gentle ocular massage.

However, the presence of other ocular diseases, such as age-related macular degeneration (AMD), may confound symptom improvement measured by QOL questionnaires. AMD, a leading cause of central vision loss in the United States, shares overlapping visual symptoms with DED, such as difficulty with driving, performing detailed tasks, and navigating [23–25]. Studies have shown that AMD, even in the mild and intermediate stages, significantly impairs visual function particularly in low contrast, low luminance, and glare environments [26]. For this reason we aimed to determine whether the severity of AMD impacts QOL improvements in patients treated for DED.

## Methods

This retrospective study was conducted at the Department of Ophthalmology, University of California San Diego (UCSD), Shiley Eye Institute on data from records of 203 eyes of 102 patients suffering from evaporative dry eyes who came to dry eye clinic and received thermotherapy in the form of LipiFlow or MiBoFlo. 40 eyes from 22 of these patients were included since they had dry AMD disease which was stable through the course of dry eye treatment. One of these patients was monocular with Phthisis bulbi in one eye. Three eyes were excluded from the study due to the presence of neovascular AMD (nAMD). Active nAMD is associated with fluctuating visual outcomes caused by retinal fluid accumulation and its subsequent resolution following anti-VEGF therapy. These changes in vision would confound our results, as the improvement in quality-of-life (QOL) questionnaire scores could not be attributed solely to dry eye treatment. Our study aimed to isolate the impact of dry eye treatment on QOL while ensuring that the underlying retinal pathology remained stable over the course of treatment. This stability was not achievable in eyes with active nAMD, where changes in vision due to the retinal disease and its treatment would introduce variability unrelated to dry eye therapy. Institutional Review Board approval from UCSD was obtained for the review of patients’ charts and images (UCSD IRB #120516). Scores for quality-of-life assessments on two standardized questionnaires were recorded before treatment and 6-months after treatment using the Ocular Surface Disease Index (OSDI) and the Standard Patient Evaluation of Eye Dryness (SPEED) questionnaires [27].

The SPEED questionnaire was scored between 0 to 28, adding scores from the frequency of symptoms on a 0–3 scale and severity of symptoms on a 0–4 scale. The symptoms assessed in the standardized questionnaire included:1. dryness/ grittiness/ scratchiness; 2. soreness/ irritation; 3. burning/ watering and 4. eye fatigue.

In the OSDI questionnaire, the frequencies of dry eye symptoms were assessed in items 1–5, the effect on daily living tasks in items 6–9 and environmental conditions that may trigger the symptoms in items 10–12. Each question was graded on a scale from 0 to 4. The total OSDI score was calculated by multiplying the sum of scores by 25 and dividing by the number of questions answered [27].

Total scores ranged from 0 to 100, with higher scores indicating worse dry eye symptoms for both questionnaires. Items 6–9 included reading, driving at night, working on a computer/ ATM and watching TV. Due to their direct relation to visual functioning, these were the ones we suspected would be affected by dry AMD disease. Hence, the 6-month post- treatment scores were subtracted from the pretreatment scores for total OSDI scores, SPEED scores and OSDI items 6–9 scores.

The difference between visual acuity before treatment and 6 months after treatment was also recorded. Snellen visual acuity was converted to ETDRS letters to facilitate calculation.

For dry AMD grading, color fundus photographs and raster Optical Coherence Tomography (OCT) scans were analyzed. These were obtained on the on the Optos Ultra-widefield (UWF™) Retinal Imaging Device and Heidelberg Spectralis imaging as part of routine evaluation.

Dry AMD was classified as early, intermediate, and late. Early AMD was defined by presence of numerous small (<63 microns) or intermediate (≥63 to <125 microns) drusen. Intermediate AMD was defined as either extensive drusen of small or intermediate size, or any drusen of large size (≥125 microns). Late AMD was defined by the presence of geographic atrophy [28].

The color fundus photographs and OCT scans pretreatment were compared with those taken 6-months after treatment to ensure no concurrent retinal changes occurred that could potentially confound the results of our study.

### Statistical analysis

We first classified eyes into three groups based on the severity of AMD: early, intermediate, and late. Changes in SPEED, and OSDI (including 6–9) questionnaire scores before and after treatment were compared among these groups using two statistical approaches. First, a multiple generalized linear mixed model was performed, with the change in SPEED or OSDI scores as the response variable, pre-treatment SPEED or OSDI as a covariate, and AMD stage as the group variable, while adjusting for age and sex. Since most patients had two eyes receiving treatment, patient identification was included as a random effect in the model. Second, given the small sample size of the study, we also performed a one-way ANOVA on ranks to compare changes in SPEED and OSDI scores across AMD severity stages. To further explore the relationship between AMD severity and treatment outcomes, we reclassified eyes into two groups: early AMD and non-early AMD and conducted a similar one-way ANOVA test on ranks for these groups. Statistical significance was defined as p < 0.05, and all analyses were conducted using the JMP software platform (JMP®, Version <16>. SAS Institute Inc., Cary, NC, 1989–2023).

## Results

The average age of patients in our study was 75.9 years. A total of 26, 9, and 5 eyes in our study group had early dry, intermediate and late dry AMD respectively.

For each of the eyes in our study, the retinal disease was stable over the 6-month study period. This stability was demonstrated by macular OCT findings, which showed no changes when compared between pretreatment and 6 months post-treatment.

The mean (± SD) visual acuity, measured in ETDRS letters, was 66.6 ± 22.2 at pretreatment and 66.3 ± 22.4 at 6 months post-treatment. There was no significant change in visual acuity over the treatment period.

All treated eyes had statistically significant improvement (p<0.01) in QOL scores across all the questionnaires studied (Table 1).

**Table 1:** Overall improvement with dry eye treatment across all groups.

Score improvement in all AMD patients	Score improvement (mean±Standard deviation)	p- value
OSDI	14.6±17	<0.001
OSDI 6–9	2.0±2.7	<0.001
SPEED	6.2±5.6	<0.001

**Fig 1:**
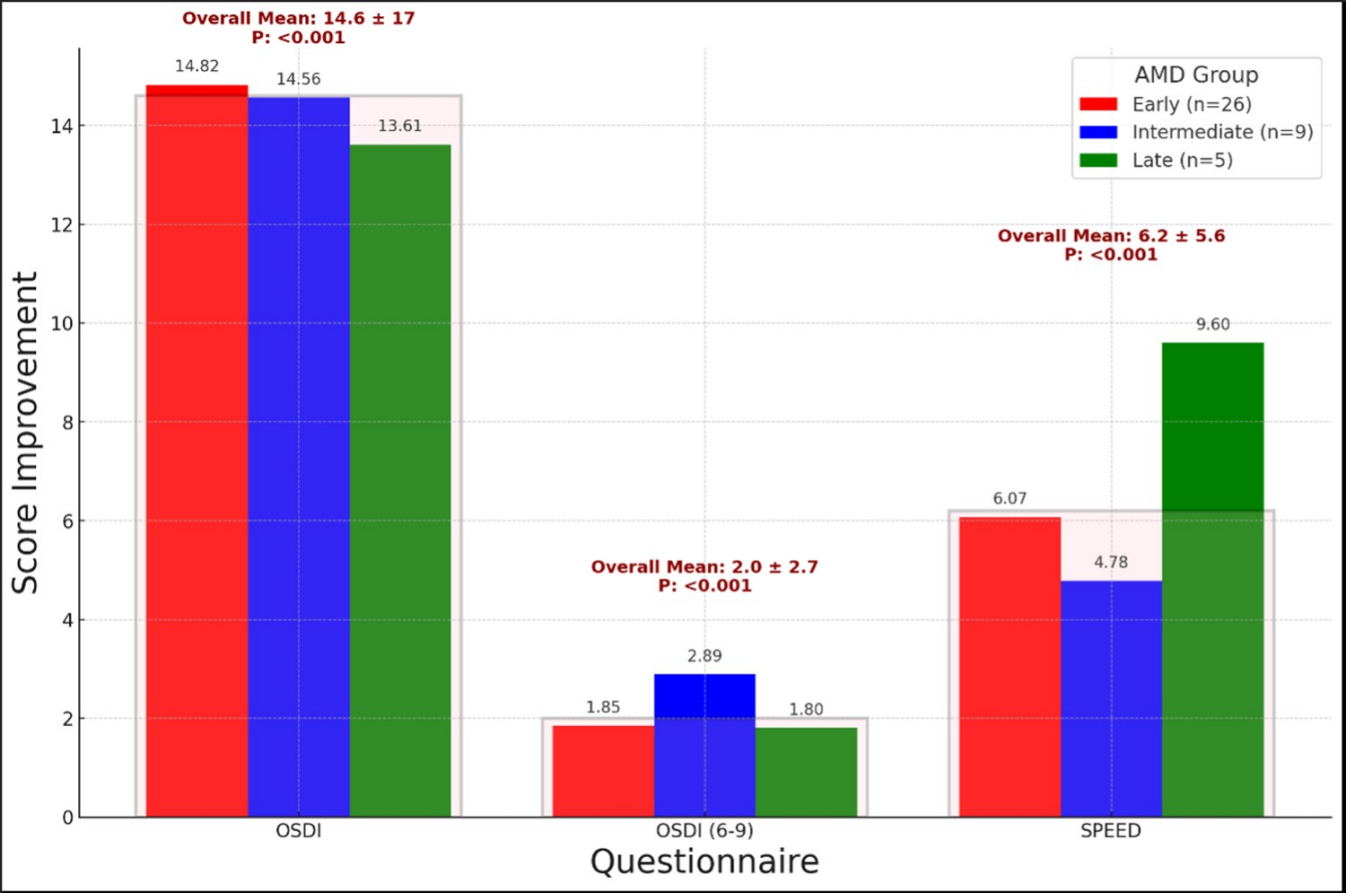
Improvements in dry eye symptoms across AMD subgroups.

For the early dry AMD group, the mean pre-treatment total OSDI score, OSDI items 6–9 score and SPEED scores were 47.57, 6.58 and 17.57 respectively. For the intermediate dry AMD group, the mean scores were 47.66, 8 and 12.11 respectively. For the late dry AMD group, the scores were 71.11, 10.2 and 18.6 respectively. The mean improvement in total OSDI score with treatment (pretreatment minus 6-month post treatment) was 14.82, 14.56, and 13.61 for early, intermediate, and late dry AMD patients whereas the improvement in OSDI scores for items 6 to 9 was 1.85, 2.89 and 1.8 for the respective groups. The mean improvement for SPEED scores with treatment (pretreatment minus 6-month post treatment) for the patients was 6.07, 4.78, and 9.6 for early, intermediate, and late dry AMD. (see Table 2). No significant difference was found between the score improvements in early, intermediate, or late dry AMD groups at p<0.05 (Table 2), showing that QOL improved with dry eye treatment irrespective of the severity of dry AMD (see Fig 1).

**Table 2:** Change in dry eye questionnaire scores with 3 AMD groups.

Change (Δ) between pretreatment and 6-month post treatment of:	Early dry AMD (N = 26)	Intermediate dry AMD (N = 9)	Late dry AMD (N = 5)	P-value for difference between early, intermediate and dry AMD groups)
Improvement in score on OSDI questionnaire	14.82	14.56	13.61	0.51
Improvement in score on OSDI items 6–9	1.85	2.89	1.8	0.07
Improvement in score on SPEED questionnaire	6.07	4.78	9.6	0.16

This bar chart illustrates the score improvement across three AMD subgroups (Early, Intermediate, and Late AMD) following dry eye treatment. It compares improvements for the OSDI, OSDI (6–9), and SPEED questionnaires. Overlay Boxes highlight the overall mean improvement, standard deviation (SD), and statistical significance (p-value) for all AMD classes combined. The translucent boxes visually encompass all three AMD subgroups for each questionnaire. Significant improvements (p < 0.001) are observed across all groups, reflecting the positive impact of dry eye treatment regardless of AMD stage.

It should be noted that a higher score in the SPEED as well the OSDI questionnaire indicates worse subjective symptoms of dry eye. Thus, a negative value obtained when subtracting the 6-month post-treatment scores with pretreatment scores indicated improvement of DED. All the mean differences in scores we studied after treatment were improvements, meaning they were all negative scores.

To improve the robustness of our study findings, we conducted an additional analysis that classified the eyes into early and non-early AMD groups. Eyes with intermediate and late dry AMD were classified together and compared to eyes with early dry AMD for this analysis.

For the early dry AMD group, the mean total OSDI score, item 6–9 OSDI score, and SPEED score were 47.57, 6.58 and 17.57 respectively. For the non-early dry AMD group, these mean scores were 56.04, 8.78 and 14.42 respectively.

The total OSDI score improvement 6 months after treatment were 14.82 and 14.22 while OSDI 6–9 item score improvement were 1.85 and 2.5 respectively for the early and non-early AMD groups. The SPEED questionnaire score improvement for the respective groups were 6.07 and 6.5.

Like the previous analysis with three groups, there was no significant difference between improvement of total OSDI, OSDI 6–9 or SPEED question scores for the early and non-early dry AMD groups (p<0.05). Here again, dry eye treatment improved the symptomatic outcomes for all treated patients irrespective of the grade of dry AMD disease.

## Discussion

Both AMD and dry eye disease contribute to visual symptoms including visual blurring, compromised night driving, reading speed and comprehension. Our study demonstrates that visual functioning improved following dry eye treatment, even in the presence of different stages of AMD pathology. It is logical that vision QOL improves across all three subgroups of AMD and that there seems to be no difference between the ability to improve QOL between AMD stages.

We observed a mean improvement of 6.2 in the SPEED questionnaire scores and 14.6 in the OSDI scores across all stages of AMD, however, there was only a small difference among the AMD stages. Since no significant differences were detected between early, intermediate, and late AMD groups, our findings suggest that dry eye treatment provides meaningful benefits to patients regardless of retinal disease stage. Separating the subjects into two groups—early and non-early AMD—provided a valuable supportive analysis, further demonstrating that the stage of AMD did not impact the response to dry eye management.

Our study is under powered to assess magnitude of QOL improvement after treatment across these subgroups. Indeed, a power analysis shows that a much larger study would be required with approximately 1000 eyes subdivided equally in each of the three subgroups to show if one AMD group had improvement more than the others.

It is possible that even with a larger sample size, our findings would retain the clinical significance of dry eye treatment in improving QOL across this population. Future research should aim to address these limitations through prospective study designs with larger, more diverse populations and a combination of subjective and objective outcome measures. Additionally, exploring the interplay between other retinal diseases and dry eye treatment outcomes could further advance our understanding of holistic eye care, particularly in populations with complex ocular comorbidities.

The most common form of dry eye treatment has traditionally been the use of topical lubricants. However, barriers such as the need for daily application, intolerance to preservatives, reduced dexterity in elderly individuals, and recurring costs can limit their lubricant effectiveness as does the short half-life of most lubricants instilled onto the ocular surface [29–33]. Therapies based on thermal expression of lipids from meibomian glands improves the physiology of the tear film and reduces lipid layer dysfunction and may eliminate or reduce the need for frequent eye drop use. Thus, their acceptance by patients is also likely to be better.

While our study demonstrates the benefits of treating dry eye disease in patients with AMD, several limitations should be acknowledged. First, the retrospective nature of the study may have introduced selection bias due to reliance on available clinical records and completed questionnaires. Second, subjective assessments like SPEED and OSDI are susceptible to patient perception and recall bias. Finally, the relatively small sample size limits the ability to determine differences amongst AMD groups.

Our study is unique in examining the relationship between AMD severity and the outcomes of dry eye treatment using validated quality-of-life (QOL) questionnaires, such as SPEED and OSDI. While prior research has demonstrated the benefits of managing ocular surface disease, none have investigated these effects in patients with coexisting retinal pathologies. 22 Our study demonstrates that improvements in QOL with dry eye treatment occur consistently across all stages of AMD. These findings highlight the importance of addressing treatable ocular conditions, even in the presence of advanced retinal disease.

Since both AMD and dry eye disease tend to affect the elderly population, it is common for AMD patients presenting to retina clinics to also have coexisting dry eye disease. Our results suggest that managing a patient’s dry eye can lead to meaningful improvements in vision related QOL, even in the presence of AMD. Thus, reduction in central vision by AMD does not preclude the need to treat dry eye and such treatment may enhance patient QOL and satisfaction.

## References

[1] Stapleton F., Alves M., Bunya V.Y., Jalbert I., Lekhanont K., Malet F., et al. TFOS DEWS II Epidemiology Report. Ocul. Surf. 2017;15:334–365. doi: 10.1016/j.jtos.2017.05.003 28736337

[2] Verjee MA, Brissette AR, Starr CE. Dry Eye Disease: Early Recognition with Guidance on Management and Treatment for Primary Care Family Physicians. Ophthalmol Ther. 2020 Dec;9(4):877–888. doi: 10.1007/s40123-020-00308-z Epub 2020 Oct 22. ; PMCID: PMC7708574. 33090327 PMC7708574

[3] Karakus S, Mathews PM, Agrawal D, Henrich C, Ramulu PY, Akpek EK. Impact of Dry Eye on Prolonged Reading. Optom Vis Sci. 2018 Dec;95(12):1105–1113. doi: 10.1097/OPX.0000000000001303 . 30439719

[4] Guo OD LW, Akpek E. The negative effects of dry eye disease on quality of life and visual function. Turk J Med Sci. 2020 Nov 3;50(SI-2):1611–1615. doi: 10.3906/sag-2002-143 ; PMCID: PMC7672346. 32283910 PMC7672346

[5] Golden MI, Meyer JJ, Patel BC. Dry Eye Syndrome. 2023 Apr 3. In: StatPearls [Internet]. Treasure Island (FL): StatPearls Publishing; 2024 Jan–. . 29262012

[6] Dartt DA, Willcox MD. Complexity of the tear film: importance in homeostasis and dysfunction during disease. Exp Eye Res. 2013 Dec;117:1–3. doi: 10.1016/j.exer.2013.10.008 ; PMCID: PMC4225770. 24280033 PMC4225770

[7] Bron AJ, Tiffany JM, Gouveia SM, Yokoi N, Voon LW. Functional aspects of the tear film lipid layer. Exp Eye Res. 2004 Mar;78(3):347–60. doi: 10.1016/j.exer.2003.09.019 . 15106912

[8] Donthineni PR, Doctor MB, Shanbhag S, Kate A, Galor A, Djalilian AR, et al. Aqueous-deficient dry eye disease: Preferred practice pattern guidelines on clinical approach, diagnosis, and management. Indian J Ophthalmol. 2023 Apr;71(4):1332–1347. doi: 10.4103/IJO.IJO_2808_22 ; PMCID: PMC10276701. 37026265 PMC10276701

[9] Findlay Q, Reid K. Dry eye disease: when to treat and when to refer. Aust Prescr. 2018 Oct;41(5):160–163. doi: 10.18773/austprescr.2018.048 Epub 2018 Oct 1. ; PMCID:PMC6202299. 30410213 PMC6202299

[10] Kloosterboer A, Dermer HI, Galor A. Diagnostic tests in dry eye. Expert Rev Ophthalmol. 2019;14(4–5):237–246. doi: 10.1080/17469899.2019.1657833 Epub 2019 Aug 29. ; PMCID:PMC6812581. 31649745 PMC6812581

[11] Walt JG, Rowe MM, Stern KL. Evaluating the functional impact of dry eye: the Ocular Surface Disease Index [abstract]. Drug Inf J. 1997;311436.

[12] Asiedu K. Rasch Analysis of the Standard Patient Evaluation of Eye Dryness Questionnaire. Eye Contact Lens. 2017 Nov;43(6):394–398. doi: 10.1097/ICL.0000000000000288 . 27341091

[13] Grubbs JR Jr, Tolleson-Rinehart S, Huynh K, Davis RM. A review of quality of life measures in dry eye questionnaires. Cornea. 2014 Feb;33(2):215–8. doi: 10.1097/ICO.0000000000000038 ; PMCID: PMC4201928. 24326332 PMC4201928

[14] Ngo W, Situ P, Keir N, Korb D, Blackie C, Simpson T. Psychometric properties and validation of the Standard Patient Evaluation of Eye Dryness questionnaire. Cornea. 2013 Sep 1;32(9):1204–10. doi: 10.1097/ICO.0b013e318294b0c0 23846405

[15] Pucker AD, Ng SM, Nichols JJ. Over the counter (OTC) artificial tear drops for dry eye syndrome. Cochrane Database Syst Rev. 2016 Feb 23;2(2):CD009729. doi: 10.1002/14651858.CD009729.pub2 ; PMCID: PMC5045033. 26905373 PMC5045033

[16] Srinivasan S, Williams R. Propylene Glycol and Hydroxypropyl Guar Nanoemulsion—Safe and Effective Lubricant Eye Drops in the Management of Dry Eye Disease. Clin Ophthalmol. 2022 Oct 10;16:3311–3326. doi: 10.2147/OPTH.S377960 ; PMCID: PMC9553314. 36237486 PMC9553314

[17] Jerkins G, Greiner JV, Tong L, Tan J, Tauber J, Mearza A, et al. A Comparison of Efficacy and Safety of Two Lipid-Based Lubricant Eye Drops for the Management of Evaporative Dry Eye Disease. Clin Ophthalmol. 2020 Jun 18;14:1665–1673. doi: 10.2147/OPTH.S256351 ; PMCID: PMC7308126. 32606581 PMC7308126

[18] Pucker AD, Yim TW, Rueff E, Ngo W, Tichenor AA, Conto JE. LipiFlow for the treatment of dry eye disease. Cochrane Database Syst Rev. 2024 Feb 5;2(2):CD015448. doi: 10.1002/14651858.CD015448.pub2 ; PMCID: PMC10840070. 38314898 PMC10840070

[19] Li S, Yang K, Wang J, Li S, Zhu L, Feng J, et al. Effect of a novel thermostatic device on meibomian gland dysfunction: a randomized controlled trial in Chinese patients. Ophthalmology and Therapy. 2022 Feb 1:1–0. doi: 10.1007/s40123-021-00431-5 34822140 PMC8770768

[20] Gomez ML, Jung J, Gonzales DD, Shacterman S, Afshari N, Cheng L. Comparison of manual versus automated thermal lid therapy with expression for meibomian gland dysfunction in patients with dry eye disease. Sci Rep. 2024 Sep 27;14(1):22287. doi: 10.1038/s41598-024-72320-3 ; PMCID: PMC11437139. 39333153 PMC11437139

[21] Hernández-Martínez N, Fernández-Vizcaya O, Pacheco-Del Valle C, Velasco-Ramos R, Babayán- Sosa A, Alegría-Gómez E, et al. Comparison of a thermoelectric heat device, tea tree oil shampoo eyelid scrubs and conventional treatment in elderly patients with meibomian gland dysfunction. Revista Mexicana de Oftalmología. 2019 Mar 21;92(6):278–85.

[22] Gomez M. L., Afshari N. A., Gonzalez D. D., & Cheng L. (2022). Effect of Thermoelectric Warming Therapy for the Treatment of Meibomian Gland Dysfunction. American journal of ophthalmology, 242, 181–188. doi: 10.1016/j.ajo.2022.06.013 35764104

[23] Fleckenstein M., Keenan T.D.L., Guymer R.H. et al. Age-related macular degeneration. Nat Rev Dis Primers 7, 31 (2021). doi: 10.1038/s41572-021-00265-2 33958600 PMC12878645

[24] Keenan TDL, Cukras CA, Chew EY. Age-Related Macular Degeneration: Epidemiology and Clinical Aspects. Adv Exp Med Biol. 2021;1256:1–31. doi: 10.1007/978-3-030-66014-7_1 . 33847996 PMC13400192

[25] Wood JM, Black AA, Mallon K, Kwan AS, Owsley C. Effects of Age-Related Macular Degeneration on Driving Performance. Invest Ophthalmol Vis Sci. 2018 Jan 1;59(1):273–279. doi: 10.1167/iovs.17-22751 ; PMCID: PMC5770181. 29340641 PMC5770181

[26] Barteselli G., Gomez M., Doede A. et al. Visual function assessment in simulated real-life situations in patients with age-related macular degeneration compared to normal subjects. Eye 28, 1231–1238 (2014). doi: 10.1038/eye.2014.189 25081294 PMC4194345

[27] Schiffman RM, Christianson MD, Jacobsen G, Hirsch JD, Reis BL. Reliability and validity of the Ocular Surface Disease Index. Arch Ophthalmol. 2000 May;118(5):615–21. doi: 10.1001/archopht.118.5.615 . 10815152

[28] Coleman HR, Chan CC, Ferris FL 3rd, Chew EY. Age-related macular degeneration. Lancet. 2008 Nov 22;372(9652):1835–45. doi: 10.1016/S0140-6736(08)61759-6 ; PMCID: PMC2603424. 19027484 PMC2603424

[29] Lanzl I, Kaercher T. Konservierte Augentropfen und Adhärenz in der augenärztlichen Praxis [Preservative-containing eye drops and adherence in ophthalmological practice]. Ophthalmologe. 2012 Nov;109(11):1087–92. German. doi: 10.1007/s00347-012-2641-9 . 23179814

[30] Agarwal P, Craig JP, Rupenthal ID. Formulation Considerations for the Management of Dry Eye Disease. Pharmaceutics. 2021 Feb 3;13(2):207. doi: 10.3390/pharmaceutics13020207 ; PMCID: PMC7913303. 33546193 PMC7913303

[31] Winfield AJ, Jessiman D, Williams A, Esakowitz L. A study of the causes of non-compliance by patients prescribed eyedrops. Br J Ophthalmol. 1990 Aug;74(8):477–80. doi: 10.1136/bjo.74.8.477 ; PMCID: PMC1042177. 2390523 PMC1042177

[32] Colomé-Campos J, Martínez-Salcedo I, Martorell-Hallado MC, Romero-Aroca P. Evaluación objetiva de la aplicación de colirios en personas mayores de 65 años [Objective evaluation of applying eye drops by elderly patients]. Arch Soc Esp Oftalmol. 2014 May;89(5):177–81. Spanish. doi: 10.1016/j.oftal.2014.02.008 Epub 2014 Apr 17. . 24746444

[33] Waduthantri S, Yong SS, Tan CH, Shen L, Lee MX, Nagarajan S, et al. Cost of dry eye treatment in an Asian clinic setting. PLoS One. 2012;7(6):e37711. doi: 10.1371/journal.pone.0037711 Epub 2012 Jun 11. ; PMCID: PMC3372539. 22701577 PMC3372539

